# Sarcomatoid Carcinoma of the Urinary Bladder Treated with Adjuvant Radiotherapy: A Case Report

**DOI:** 10.4137/ccrep.s3126

**Published:** 2009-07-30

**Authors:** Cem Onal, Berrin Pehlivan, Nebil Bal, Erkan Topkan, Ferhat Kilinc, Savas Topuk

**Affiliations:** 1 Assistant Professor, Department of Radiation Oncology, Baskent University Medical Faculty, Adana, Turkey.; 2 Associate Professor, Department of Pathology, Baskent University Medical Faculty, Adana, Turkey.; 3 Associate Professor, Department of Urology, Baskent University Medical Faculty, Adana, Turkey.; 4 Resident, Department of Radiation Oncology, Baskent University Medical Faculty, Adana, Turkey. Email: hcemonal@hotmail.com

**Keywords:** bladder, sarcomatoid carcinoma, surgery, radiotherapy

## Abstract

Sarcomatoid carcinoma is a rare tumor of the urinary bladder accounting for less than 0.5% of all primary urinary bladder tumors. Since the patients were presented with large tumor with extended stages, outcome was found to be poor. In order to improve local control, adjuvant local treatment may be practical. We report a rare case with sarcomatoid carcinoma of the urinary bladder diagnosed with immunuhistochemical (IHC) study and treated with 3D-conformal radiotherapy (3DCRT) post-operatively. A 55-year old female patient complained about painless hematuria for 2 months. Computed tomography of the pelvic region revealed tumor and wall thickening at the left posterolateral side of the bladder. Total cystectomy with lymph node dissection and total abdominal hysterectomy and bilateral salphingo-oopherectomy was performed and histopathological and immunohistochemical findings strongly correlate with sarcomatoid carcinoma. The patient was treated with 3D conformal radiotherapy (3DCRT) with a total dose of 59.4 Gy with 1.8 Gy fractional daily doses. Patient was alive without any local recurrence and distant metastasis 10 months after surgery.

## Introduction

Sarcomatoid carcinoma (SC) is a rare entity arising in the urinary bladder accounting for less than 0.5% of all primary urinary bladder tumors.^1^ SC is defined as a variant of urothelial carcinoma displaying a prominent spindle cell or malignant fibrous histiocytoma-like growth pattern.^2^,^3^ The etiology and clinical manifestation of this tumor is still unknown because of its rarity.

Outcome was poor in patients with SC, because the patients were presented with large tumor with extended stages. Since SC is locally aggressive tumor, adjuvant local treatment may improve local control. Although some reports of SC has been reported mostly from the Japanese literature; we report a rare case with SC of the urinary bladder diagnosed with immunuhistochemical (IHC) study and treated with 3D-conformal radiotherapy (3DCRT) post-operatively.

## Case Report

A 55-year old female patient complained about painless hematuria for 2 months, increasing in last 5 days. Her past history and family history were not remarkable except for smoking (3–4 cigarettes per day for almost 35 years), and a weight loss of 5 kg within last 1 month. In physical examination, no pathological findings were found. The blood chemistry was within normal limits except for mild anemia with a hemoglobin level of 11.5 g/dL, and urine was macroscopically bloody.

**Figure 1:**
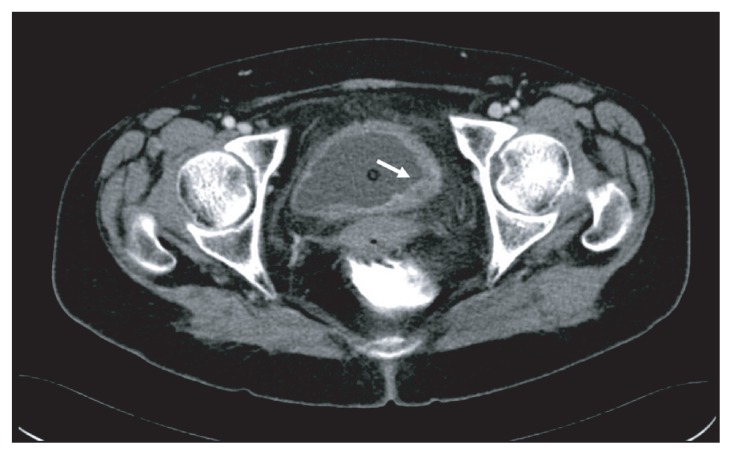
Pelvic computerized tomography revealed a 3.5 cm tumor at the left lateral wall of the bladder (arrow).

**Figure 2:**
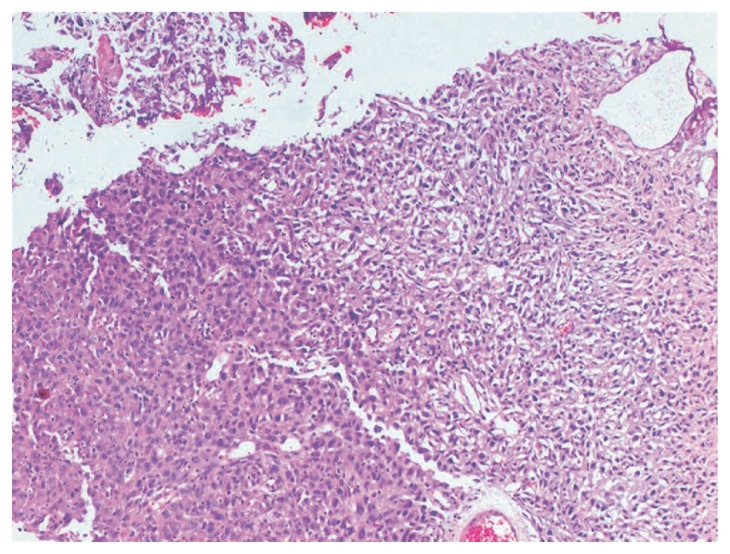
Tumor have an epitheloid and sarcomatoid areas (H&E x 200).

**Figure 3:**
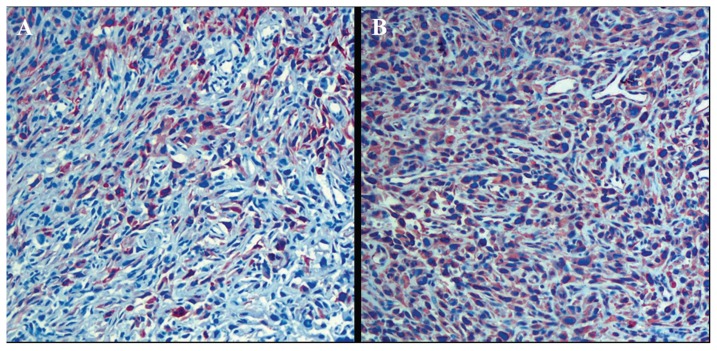
(**A**) Diffuse vimentin positivity of tumor cells (Vimentin x 200), (**B**) and cytokeratin positivity of bladder (Cytokeratin x 200).

Computed tomography (CT) of the pelvis demonstrated a 2.5 cm tumor at the left posterolateral wall and a 3.5 cm tumor at the left lateral wall of the bladder (Fig. 1). Bone scintigraphy and thoracoabdominal CT revealed no metastatic lesions. In cystoscopy, a 2.5-cm-hemorrhagic tumor locating at the posterior side of the left orifice, not obstructing the passage was demonstrated. Total cystectomy with lymph node dissection and total abdominal hysterectomy and bilateral salphingo-oopherectomy was performed. Macroscopically, 3.5 and 2.5 cm tumors infiltrating the adipose tissue at the lateral wall was seen. Microscopically, this tumor infiltrates all layers of bladder wall. These tumors were composed of sarcomatoid components and also carcinomatous components with high grade cellular atypia, and spindle cells and epihleloid cells were scattered within the tumor (Fig. 2). Immunohistochemically, tumor was stained prominently with vimentin (Fig. 3a), and focally with keratin (Fig. 3b). Desmin was negative. Histopathological findings revealed a high grade sarcomatoid carcinoma of the urinary bladder. The patient was treated with 3D conformal radiotherapy (3DCRT) with a total dose of 59.4 Gy with 1.8 Gy fractional daily doses, one month after surgery. Patient was alive without any local recurrence and distant metastasis 10 months after surgery.

## Discussion

Tumor constituting both epithelial malignant components and non-epithelial malignant components is extremely rare. Torenbeek et al reported that of 4191 patients with bladder carcinoma, the incidence of SC was only 0.3%,^4^ while Ikegami et al reported the incidence of SC was 1.06%.^3^ Since SC of bladder is rare tumor, there is limited data regarding etiology and outcome of the SC of the bladder. We reported a rare case with SC of bladder treated with adjuvant 3DCRT, which is lacking in English literature.

The median age at diagnosis is 60–70 years with a male: female ratio of 4:1.^5^ Most patients with SC of the bladder were presented with painless hematuria, as was in our case. The typical clinical presentation of SC of the bladder is left sided submucosal mass locating at the base of the bladder around the trigone, like in our patient.

In previous reports, although it is a rare entity, various terms have been used for such tumors, such as sarcomatoid carcinoma, carcinosarcoma, spindle cell carcinoma, Mullerian tumor, metaplastic carcinoma and malignant mixed mesodermal tumor.^2^,^6^ Although the terms carcinosarcoma and sarcomatoid carcinoma have been used together, the term carcinosarcoma describes the tumor composed of both malignant epithelial and malignant soft tissue elements. Meanwhile, the term sarcomatoid carcinoma has been used to describe malignant spindle cell type tumor with epithelial differentiation.^2^ The most common sarcomatous elements are chondrosarcoma, leiomyosarcoma, fibrosarcoma and rhabdomyosarcoma, while the epithelial components consist of transitional cell carcinoma, small cells carcinoma, squamous carcinoma and other mixed forms.^4^ Although it is very important and desirable to clarify the histogenetic differences between these two variants, histological findings are of no definite clinical significance as yet. In order to make accurate diagnosis, IHC study is of particular value in these tumors.^2^,^3^,^6^,^7^ In our case, histopathologic findings demonstrated that sarcomatoid component consisted of spindle cells and epithleloid cells were scattered within the tumor. Also IHC findings were tumor stained prominently with vimentin, and focally with keratin, suggesting that the tumor was derived from an epithelial origin.

There is limited data about clinical outcome of SC of the urinary bladder only from case reports; however, it is difficult to assess all cases due to the vague diagnostic standards used in each report. Appropriate clinical behavior is not clear for such rare diseases; but very aggressive behavior and usually loco-regional extension are the striking features of this neoplasm.^8^ Total cystectomy is the preferred treatment as for an invaded bladder cancer, often followed by radiotherapy or chemotherapy.^1^,^6^,^7^,^9^ However the effectiveness of these treatments is not still well-known due to the varying results of each case. In a study with 26 patients with SC of the bladder, Lopez-Beltran pointed out that the median survival was 10 months.^2^ In a case series of three patients, Sejima et al demonstrated that 2 of 3 patients were died 2 and 3 months after radical cystectomy due to local recurrence.^10^

In order to achieve better local control, adjuvant RT with modern techniques using either 3DCRT or IMRT should be useful. The results of the current case suggest that adjuvant 3DCRT with higher doses is well-tolerated and might be capable of improving the local control and relatively overall survival. Further study with more cases and experience may provide great value, pathologically and clinically.
